# Pressure-Relieving Effect of Different Insole Top Covers in People with Diabetes at High Risk of Foot Ulceration

**DOI:** 10.3390/s24175549

**Published:** 2024-08-27

**Authors:** Sicco A. Bus, Tessa E. Busch-Westbroek, Jan Pulles, Tim van Dun, Ghizella Szabo, Dario H. Lacorte, Dannick Luckson, Jaap J. van Netten

**Affiliations:** 1 Department of Rehabilitation Medicine, Amsterdam University Medical Center, University of Amsterdam, Meibergdreef 9, 1105 AZ Amsterdam, The Netherlandsj.j.vannetten@amsterdamumc.nl (J.J.v.N.); 2 Program Rehabilitation & Development, Amsterdam Movement Sciences Research Institute, van der Boechorststraat 9, 1081 BT Amsterdam, The Netherlands; 3 Livit OttoBock Care, Kabelweg 40, 1060 JA Amsterdam, The Netherlands; 4 Voetencentrum Wender, Sabina Klinkhamerweg 10, 7555 SK Hengelo, The Netherlands; 5 Department of Human Movement Sciences, Vrije Universiteit Amsterdam, van der Boechorststraat 7, 1081 BT Amsterdam, The Netherlands

**Keywords:** diabetic foot, footwear, biomechanical sensors, offloading, materials, foam

## Abstract

Pressure-relieving footwear helps prevent foot ulcers in people with diabetes. The footwear design contributes to this effect and includes the insole top cover. We aimed to assess the offloading effect of materials commonly used as insole top cover. We measured 20 participants with diabetes and peripheral neuropathy for in-shoe peak pressures while walking in their prescribed footwear with the insole covered with eight different materials, tested in randomized order. Top covers were a 3 mm or 6 mm thick open or closed-cell foam or a 6 mm thick combination of open- and closed-cell foams. We re-assessed pressures after one month of using the top cover. Peak pressures were assessed per anatomical foot region and a region of interest (i.e., previous ulceration or high barefoot pressure). Walking comfort was assessed using a 10-point Likert scale. Mean peak pressure at the region of interest varied between 167 (SD:56) and 186 (SD:65) kPa across top covers (*p* < 0.001) and was significantly higher for the 3 mm thick PPT than for four of the seven 6 mm thick top covers. Across 6 mm thick top covers, only two showed a significant peak pressure difference between them. Over time, peak pressures changed non-significantly from −2.7 to +47.8 kPa across top cover conditions. Comfort ratings were 8.0 to 8.4 across top covers (*p* = 0.863). The 6 mm thick foams provided more pressure relief than the 3 mm thick foam during walking in high-risk people with diabetes. Between the 6 mm thick foams and over time, only small differences exist. The choice of which 6 mm thick insole top cover to use may be determined more by availability, durability, ease of use, costs, or hygienic properties than by superiority in pressure-relief capacity.

## 1. Introduction

In people with diabetes-related foot disease, a plantar foot ulcer most commonly occurs as a result of repetitive high plantar peak pressures during ambulatory activities in the presence of loss of protective sensation due to peripheral neuropathy [1]. Reduction of these high peak pressures, or offloading, is therefore an important aim to help prevent plantar foot ulcers, in particular when people are in remission after healing from such an ulcer (International Working Group on the Diabetic Foot (IWGDF) risk 3) [2]. Custom-made (therapeutic) footwear aims to reduce high pressures by redistributing pressure from high risk locations to lower risk locations and is recommended in international guidelines for ulcer prevention [3]. As part of the construction of such footwear, different insole and shoe design features have shown to contribute to relieving plantar peak pressure. This includes the use of a custom-made insole, a metatarsal pad or bar, medial arch support, a rocker-outsole configuration, and increased stiffness of the shoe outsole [4,5,6,7,8,9,10,11]. These design features are used in clinical practice and are included in state-of-the-art footwear design protocols for people with diabetes at high risk of plantar foot ulceration [12].

The top cover of the insole is another shoe design feature that may have an effect on the peak pressures measured during walking [13,14,15,16]. These top covers could be of single- or multi-density materials, of variable thickness, and of closed-cell and open-cell foams. Previous research showed that multi-density foams offload the plantar foot surface of people with diabetes at high ulcer risk (IWGDF risk 3) more effectively than no top cover or a leather top cover [4]. Three recent systematic reviews concluded that for other comparisons related to the top cover(s), little evidence is yet available [13,16,17]. This may be the reason that many different types and combinations of materials are used in clinical footwear practice and likely based just as much on availability, ease of use, hygienic properties (i.e., anti-bacterial and anti-fungal), and/or costs than known effectiveness. A better understanding of the comparative effect of different top covers would provide valuable information to improve custom-made footwear design for people at high foot ulcer risk and to help decision making for the insole top cover to best offload the at-risk foot. Therefore, the aim of this study was to compare the effect of various single- and multi-density top cover materials commonly used in clinical footwear practice on peak plantar pressure and comfort while walking in people with diabetes at high risk of foot ulceration. We first aimed to assess this effect cross-sectionally with newly provided insole top covers and subsequently over a one-month period to assess wearing effects of the top covers.

## 2. Methods

### 2.1. Study Design

This was a combination of a cross-sectional study (part 1) and a before–after prospective study with a one-month follow-up (part 2).

### 2.2. Participants

Twenty persons with diabetes who had loss of protective sensation due to peripheral neuropathy were recruited from the outpatient diabetic foot clinic of Amsterdam UMC, The Netherlands. All participants were either IWGDF risk 2, i.e., with foot deformity in addition to loss of protective sensation, or IWGDF risk 3, i.e., history of a foot ulcer [18]. Participants with an ulcer, Charcot midfoot deformity, an amputation of more than two toes or the hallux, or inability to walk (unaided) repeatedly for at least 20 m (as observed by the investigator) were excluded. The study protocol was approved by the medical ethics committee of Amsterdam UMC (METC 2019_130, NL68629.018.19). All participants provided written informed consent prior to the start of the study. The study sample of 20 participants was a convenience sample; no statistical-based sample size calculation was performed. The sample was considered large enough for the purpose of this study, based on other published studies on similar interventions using a similar sample size [10,11].

### 2.3. Insole Top Cover Conditions

Each insole top cover was tested in the own, prescribed footwear of the participant, which was a custom-made insole worn in a custom-made or extra-depth shoe. Eight insole top covers were tested in each participant for the cross-sectional study. These are commonly used insole top covers in footwear practice (see Table 1). The insole top covers consisted of single-density or multi-density open-cell and closed-cell foams, with thicknesses of 3–7 mm. Open-cell foams are from polyurethane and contain cells that are not completely sealed and allow air to fill the space, meaning it can be easily compressed and then naturally recovers to its original shape. Closed-cell foams are from polyethylene and have uniform, interlocking cells that are sealed, preventing air from passing through and making the foam more rigid and deformable. Both foams are used for shock absorption. When combined, the closed-cell foam is the one on top in contact with the foot. The insole top covers were glued to the base of the insole by a certified and experienced orthopaedic shoe technician.

### 2.4. Measurements

At baseline visit, demographic and disease-specific data were obtained. Presence of loss of protective sensation due to peripheral neuropathy was tested using touch pressure sensation assessment with the 10 g Semmes-Weinstein 5.07 monofilament at three forefoot locations on each foot. Loss of protective sensation was considered present when the filament could not be felt at least at two locations per foot [18]. Foot deformity was assessed in non-weight-bearing condition by observation by the involved clinician (TEBW) and classified as absent, mild (i.e., pes planus, pes cavus, hammer toe, hallux valgus or limitus, lesser toe amputation), moderate (i.e., claw toe, prominent metatarsal heads, hallux rigidus, hallux, or ray amputation), and severe (i.e., pes equines or forefoot amputation) [2].

In each participant, barefoot plantar pressures were measured during walking using an EMED-X platform (Novel, Munich, Germany) that was set flush with the ground in the laboratory for clinical gait analysis of Amsterdam UMC. The EMED-X platform consists of capacitance-based sensors in a spatial resolution of four per square centimetre sampled at 70 Hz. Participants hit the platform on their second step after initiation of gait [19]. Four walking trials over the platform were conducted per foot, and average peak pressures per foot were calculated.

For study part 1 (the cross-sectional study), the insole top covers were assessed in random order, determined using self-written code (Matlab, MathWorks, Natick, MA, USA). During assessment, the participant walked along a 12 m walkway in the laboratory for clinical gait analysis or along a 30 m walkway in the hallway of the department at Amsterdam UMC. Measurements in the hallway were taken when the laboratory was occupied for other clinical gait testing. Walking speed in the laboratory was calculated from measured time between photocells positioned 5.5 m apart along the walkway. In the hallway, walking speed was calculated from measured time using a stopwatch between cones placed 20 m apart along the walkway. Because walking speed affects peak pressures, walking speed was standardized between conditions to the preferred walking speed obtained during testing of the first condition (max 5% deviation allowed). In-shoe plantar pressures during walking with each condition were measured using the pedar-X system (Novel, Munich, Germany). This system consists of measurement insoles with 99 capacitance-based sensors sampling at 50 Hz. These pedar insoles were inserted in the shoe before the shoe was put on, with that being the interface between the top cover and the foot. A minimum of 12 midgait steps per foot per condition were collected for reliable data, meaning that 2–4 walking trials were collected per condition, depending on whether measurements were done in the laboratory or in the longer hallway [20]. Directly after the pressure measurements in each condition, participants were asked to rate their shoe fit and their level of walking comfort with the top cover condition just testes. Both shoe fit and walking comfort were assessed using a 10-point Likert scale, with 1 being the lowest possible and 10 the highest possible shoe fit or walking comfort [10].

For study part 2 (the prospective follow-up study), the insoles of the left and right shoe of the participant were covered with two different and randomly selected top covers (randomization again using self-written Matlab code). Participants wore their shoes with the insole top covers for one month, after which they were re-tested for in-shoe plantar pressure in the laboratory or hallway using the same protocol as described above. Walking speed was standardized between occasions to the preferred average walking speed obtained during the first testing session (max 5% deviation allowed). After these measurements, the insoles of the left and right shoe were covered again with two different and randomly selected top covers. Participants wore their shoes with these insole top covers again for a month, after which they were tested for in-shoe plantar pressure in the laboratory or hallway, walking at standardized walking speed. Using this protocol and scheme, each insole top cover could be tested in 7–10 participants for this prospective follow-up study.

### 2.5. Data Analysis

Peak pressure distribution pictures from the in-shoe pressure measurements were masked into 10 anatomical regions using Novel Multimask Software (version 24.3.20): medial and lateral hindfoot, medial and lateral midfoot, first metatarsal head (MTH1), second and third metatarsal heads (MTH23), fourth and fifth metatarsal heads (MTH45), hallux, second and third toe (Dig23), and fourth and fifth toe (Dig45). One of these ten regions per foot was additionally selected as region of interest (ROI), being the region where the previous ulcer was located or, if no plantar ulcer history, where the highest barefoot peak pressure was present. Mean peak pressures over the steps taken were calculated for each of the 10 anatomical regions and for the ROI. For the cross-sectional study, peak pressures were averaged across the left and right foot. Other pressure parameters than peak pressure were not assessed due to high correlation with peak pressure or less evidence for clinical relevance [21].

### 2.6. Statistical Analysis

In the cross-sectional study, regional peak pressures were statistically analysed for insole top cover condition using repeated measures ANOVA with a Greenhouse–Geisser correction. Post hoc analyses with a Bonferroni adjustment were performed to test for statistically significant differences between two insole top covers. Paired-sample *t*-tests were conducted to explore differences in peak pressure before and after one-month follow-up for each of the eight insole top covers. Because each top cover was worn by only 7–10 participants, analyses were within and not between top covers; no correction for multiple testing was applied here given the explorative nature of the analyses. All descriptive and statistical analyses were performed using SPSS for windows (IBM SPSS Statistics version 22, Armonk, NY, USA). All tests were performed with a significance level of 0.05. 

## 3. Results

The baseline characteristics of the participants are summarized in Table 2. The region of interest (ROI) was the hallux in 45% of cases, MTH1 in 18%, and MTH23 in 28%. Results at the ROI were therefore mostly determined by the outcomes for these three regions.

Mean peak pressure at the ROI varied between 167 (SD:56) and 186 (SD:65) kPa across top covers and was significantly different between insole top covers (F(4.198; 163.736) = 6.728, *p* < 0.001) (Table 3). Post hoc analysis showed the 3 mm thick PPT top cover to give statistically significantly higher peak pressures compared to the 6 mm thick PPT/P-cell (mean difference 17.6 kPa; 95%CI: 4.2–31.0), 6 mm thick Astro form/Aero sorb (mean difference 19.3 kPa; 95%CI: 7.7–31.0), 6 mm thick PPT (mean difference 14.5 kPa; 95%CI: 4.7–24.4) and 6 mm thick Lunatec (mean difference 17.3 kPa; 95%CI: 3.6–31.1). Furthermore, the Vepur/Poron combination showed significantly higher peak pressures compared to the Astro form/Aero sorb top cover (mean difference 9.2 kPa; 95%CI: 0.1–18.3).

For the 10 anatomical regions, statistically significant differences in in-shoe peak pressures were also found across insole top covers, as shown in Table 3 for the five priority regions (as ROIs) and in Supplemental Table S1 for the other five regions. However, in some of the regions, the effects were quite small (effect size < 0.1 and percentage difference between highest and lowest peak pressure < 10% for all three toe regions and lateral metatarsals), with large standard deviations around the mean peak pressure per condition. In post hoc analysis, statistically significant differences were found similar to the ROI, with the 3 mm thick PPT showing significantly higher peak pressures in regions MTH1, MTH23, the medial and lateral midfoot, and the medial and lateral heel compared to several 6 mm thick top covers (Table 3 and Supplemental Table S1), with Vepur/Poron showing higher pressures in the MTH1 and region compared to several other 6 mm thick top covers (Table 3). No statistically significant differences were found between insole top covers in post hoc analyses in the hallux, while Vipod/DiaPod showed higher pressures for the lesser digits (both Dig23 and Dig45; Supplemental Table S1).

Results for the perception of participants wearing the top covers at baseline are shown in Table 4. The outcomes for the items shoe fit and walking comfort were generally high, with mean shoe fit scores ranging between 7.8 and 8.4 and for walking comfort between 8.0 and 8.4. Scores were also quite similar across top covers, with none of the differences between top covers being statistically significant on the group level (for fit: F = 1.0; *p* = 0.435, effect size = 0.056; for comfort: F = 0.458; *p* = 0.863; effect size = 0.026) or in post hoc analyses.

The in-shoe peak pressure changes after one month of wearing the insole top covers are shown in Table 5 and Supplemental Table S2. At the ROI, peak pressures for most top covers increased in one month, even up to a mean 47.8 kPa in the PPT/P-cell condition. However, none of these changes were significant (0.078 < *p* < 0.879). The effects at the ROI were mostly determined by those at the hallux (45% of ROI), which showed variable effects across top covers, with differences ranging from −37.6 kPa (6 mm thick PPT, *p* < 0.05) to +40.5 kPa (PPT/P-cell). At MTH1, absolute and relative effects across top covers over time were smaller, but for all top covers, they were in the direction of pressure relief at 1 month: −12.6 to −2.0 kPa, with none being statistically significant. Vipod/DiaPod was the only top cover that showed lower peak pressures over time in all 10 anatomical regions (−11.7 to −1.0 kPa), and Vepur/Poron showed the same in 9 out of 10 regions (−30.0 to −4.6 but showing an increase at the hallux of 19.6 kPa). The 6 mm thick PPT showed on average the largest reductions over time out of all top covers in 8 of the 10 regions (−37.6 to −4.9 kPa), with 4 showing a significant difference. The PPT/P-cell top cover showed in 9 of 10 regions small to large increases in peak pressure over time (4 regions with +28.2 to +40.5 kPa). Due to the large number of paired *t*-tests taking all regions together, the chance of a type 1 error was present (i.e., a significant difference (all between *p* < 0.01 and *p* < 0.05) not being a true change).

## 4. Discussion

We compared the in-shoe peak plantar pressures of eight different commonly used insole top covers applied in custom-made footwear, and we assessed their effect on in-shoe pressure over a one-month period. We also assessed participants’ satisfaction while walking with each insole top cover. The 6 mm thick insole top covers generally relieved peak pressure to a greater degree than the 3 mm thick open-cell foam top cover, by an average range of 14.5–19.3 kPa or 7.8–10.4% at the ROI. Across 6 mm thick top covers, only small differences up to 9.1 kPa or 5.2% were found. After one month of wearing the top cover, variable effects across top covers were found on in-shoe peak pressure, with no significant changes over time. Participant satisfaction was high and not significantly different between top covers. This seems to show that different 6 mm thick insole top covers can be used in clinical footwear practice for offloading efficacy. 

Traditionally, thinner materials have been used as top cover for insoles for people with diabetes [2]. However, a trial on the efficacy of pressure-optimized footwear clearly showed that a 6 mm thick multi-density PPT and Plastazote combination was more effective than these thinner covers [2,4], which was confirmed by another study on the pressure effects of adding a multi-density top cover [22] and also shown and summarized in three recent systematic reviews on the topic [13,16,17]. The current study also showed that the 6 mm thick insoles were more effective in offloading than the 3 mm thick one. Based on the current and these previous studies, a thickness of 6 mm therefore seems most appropriate as an insole top cover for pressure relief in custom-made footwear design for people with diabetes. While implementation of the use of these 6 mm thick top covers is slow, the current findings may help move this implementation forward. For this purpose, the type of 6 mm thick material does not seem to be a decisive factor and can be a combination of closed-cell and open-cell foams or a full layer of closed-cell or open-cell foam despite the different physical properties of these materials [14,15]. The small non-significant differences found for perception of walking give further support for the similarity between top covers; no earlier studies assessed the effects of different top covers on the perception of walking. This provides several opportunities for using different materials for the purpose of pressure relief in footwear for high-risk people with diabetes. Some materials may be easier to use (applying and removing from the insole base), while others may have better anti-bacterial or anti-fungal properties, be more available, or have a lower cost, which together may determine which insole top cover is chosen.

The effects of insole top covers over time are important to assess, as the closed-cell materials may further accommodate to the shape of the foot, likely further relieving pressure over time, but will also wear and tear, likely increasing pressure over time [2]. The explorative results, which are the first, to the best knowledge of the authors, show that for several top covers and several foot regions, relatively large pressure changes occurred over time. However, maybe because of the low sample size for this comparison, only few changes were significant in univariate analyses and would become non-significant when more rigorous correction for multiple sampling would take place. Some insole top covers, however, were consistent in providing a relief in peak pressure over time across foot regions, whereas one top cover generally showed quite large increases in peak pressure over time (Table 5). Future research on larger samples and with longer follow-up to better examine the longer-term pressure and wear-and-tear effects of insole top covers will likely further guide decision making.

### Strengths and Limitations

A strength of the study is that the results are representative of normal clinical practice, with high-risk people with diabetes being tested in their own custom-made footwear with commonly used insole top covers. Furthermore, both cross-sectional and prospective comparisons were made. The study was limited in that we did not compare with a baseline reference condition of no top cover to determine the pressure-reliving effect of using foam materials. This was because of the many conditions already tested and comparisons made with the top covers and because previous studies have already shown the beneficial effect of using foam materials over no foam use [4,13,23,24,25]. For similar reasons, not more than eight different top covers were compared, even though more (combinations of) materials exist or are possible, and we may have missed some commonly used materials and combinations in footwear practice. Furthermore, most top covers were 6 mm thick, and only one was 3 mm thick. While this may represent footwear practice, comparison with more 3 mm thick covers would have strengthened the study findings, potentially allowing more robust conclusions about the thickness effect.

No statistically based sample size calculation was conducted; a convenience sample of 20 participants was chosen, which is comparable to the sample size in other studies using similar designs and interventions [8,10,11]. Furthermore, the assessments of the effects of wearing the top cover over time were exploratory: They were limited to one month, which is shorter than the lifecycle of a top cover, and limited to 7–10 participants per each top cover that was tested. With assessments being performed at multiple time points over a longer time and with each top cover tested in a larger sample, possibly more differentiation (and statistical differences) between top covers in their effect on peak pressure may occur and help decision making. Related to this, we did not measure the number of steps taken as an indication for the cumulative load put on the insole top cover during the month of wear. Based on data from a previous trial showing a mean number of daily steps of 6397 and an adherence of 71% in similar participants [2,26], an approximate mean of 68.000 steps (per foot, with a large variation between participants expected) may have been taken by the participants with each top cover condition. A final limitation was that we could only measure normal peak pressure and not shear, even though with different materials being worn directly against the skin of the plantar foot, shear may be an important and differentiating component in the comparison between insole top covers [27].

## 5. Conclusions

In conclusion, 6 mm thick insole top covers provide more in-shoe peak pressure relief than a 3 mm thick open-cell foam top cover. Across 6 mm thick top covers, only small differences in pressure effect were found, regardless of whether they were single- or multiple-density or open- or closed-cell foam materials. The same was true for the perceived comfort with walking with the insole top covers. One month of wearing the insole top cover showed no significant changes in peak pressure compared to baseline, although peak pressure seemed to increase in some and reduce in other top covers. Therefore, different 6 mm thick insole top covers can be used in clinical footwear practice for the purpose of relieving plantar peak pressure in people with diabetes at high risk of foot ulceration. The choice of top layer may be determined more by availability, durability, hygienic properties, ease of use, costs, or sustainability than by superiority in offloading capacity. 

## Figures and Tables

**Table 1:** Insole top cover conditions and characteristics.

Top Cover		Material Brand Name	Thickness	Properties	Image	Shore A	Density (g/cm^3^)
A	Top	Plastazote^® 1^	3 mm	Closed-cell polyethylene	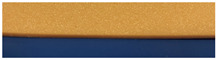	18	0.05
	Bottom	PPT^® 2^	3 mm	Open-cell polyurethane	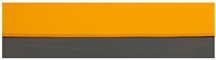	20	0.38
B	Top	P-cell^® 3^	3 mm	Closed-cell ethyl vinyl acetate	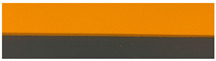	20	0.07
	Bottom	PPT^® 2^	3 mm	Open-cell polyurethane	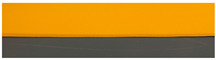	20	0.38
C	Top	Nora Aero sorb W^® 4^	3 mm	Closed-cell	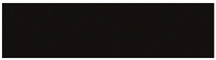	soft	0.16
	Bottom	Nora Astro form 8^® 4^	3 mm	Open-cell	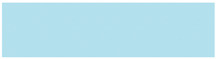	8	0.21
D		PPT^® 2^	6 mm	Open-cell polyurethane	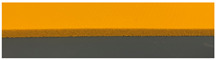	12	0.35
E		PPT^® 2^	3 mm	Open-cell polyurethane	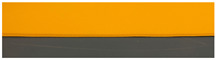	20	0.38
F		Nora Lunatec Motion^® 4^	6 mm	Closed-cell	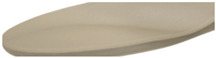	12	0.13
G	Top	Poron^® 5^	3 mm	Open-cell polyurethane	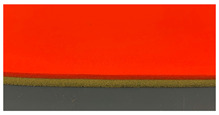	17	0.24
	Bottom	Vepur Polyurethane (PU)^® 5^	4 mm	Open-cell polyurethane		-	-
H	Top	DiaPod^® 6^	4 mm	Closed-cell	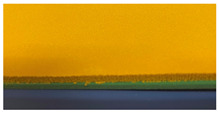	25	0.15
	Bottom	Vibram^®^ Vipod^® 6^	2 mm	Closed-cell		20	0.24

Sources/manufacturers: ^1^ Azote^®^, London, UK; ^2^ J. van Drunen, Nieuwkuijk, Netherlands; ^3^ Acor^®^ Orthopaedic, Cleveland, OH, USA; ^4^ Nora^®^ systems shoe components GmbH, Weinheim, Germany; ^5^ Bauerfeind^®^ Orthopadie GmbH, Zeulenroda, Germany; ^6^ Podartis^®^ SrL, Crocetta del Montello, Italy. Top cover G has a thickness of 7 mm but, being the only one, is referred to as a 6 mm-thick top cover in the main text.

**Table 2:** Baseline characteristics.

Participants	*n* = 20
Characteristics	
Age (years)	71 ± 7
Sex (female/male)	15% (3)/85% (17)
Diabetes type (1/2)	15% (3)/85% (17)
Diabetes duration (years)	20 ± 15
BMI (kg/m^2^)	31 ± 6
Loss of protective sensation	100% (20)
Walking speed (m/s)	1.0 ± 0.2
**Foot ulcer history**	***n* = 18**
Ulcer history	90% (18)
- Recent (<1 year)	45% (9)
Time since healing of last ulcer (months) *	15 ± 17
Foot (most recent ulcer)	
- Left	39% (7)
- Right	61% (11)
Ulcer location (most recent ulcer)	
Plantar	
- Hallux	33% (6)
- Digits	28% (5)
- Metatarsal region	17% (3)
- Midfoot	0
- Heel	5% (1)
Dorsal	17% (3)
**Feet**	***n* = 40**
ROI **	
- Hallux	45% (18)
- MTH1	18% (7)
- MTH23	28% (11)
- MTH45	5% (2)
- Medial midfoot	5% (2)
Barefoot PPP (kPa) at ROI during walking	
- Left foot (*n* = 20)	981 (239)
- Right foot (*n* = 20)	944 (285)
Foot deformities ***	
- Absent	13% (5)
- Mild	8% (3)
- Moderate	78% (31)
- Severe	3% (1)
Amputations	
- No amputation	97% (39)
- Second ray (right foot)	3% (1)

Note: Continuous data are mean ± standard deviation and discrete data are percentage (number). PPP = peak plantar pressure. MTH = metatarsal head region. *: *n* = 15, with healing date missing in *n* = 3; ** ROI = region of interest = primary region for offloading, based on most recent plantar ulcer location or location with the highest barefoot pressures; *** = foot deformity was classified as absent, mild, moderate, and severe [2]. BMI = body mass index. PPP = peak plantar pressure.

**Table 3:** Mean peak pressures (SD) at the ROI plus the five anatomical regions that were identified as a ROI for all eight insole top covers in the cross-sectional study.

Insole	Name (Bottom/Top)		ROI	Hallux	MTH1	MTH23	MTH45	Medial Midfoot
A	PPT/Plastazote, 6 mm	Mean	173.5	153.3	143.2	149.3	111.0	107.4
		SD	(55.9)	(68.9)	(40.6)	(32.8)	(31.8)	(37.8)
B	PPT/P-cell, 6 mm	Mean	168.3	149.7	137.7	148.0	114.9	107.1
		SD	(52.0)	(67.1)	(36.1)	(31.9)	(34.8)	(35.8)
C	Astro form/Aero sorb, 6 mm	Mean	166.6	148.3	138.6	149.1	110.8	104.5
		SD	(55.5)	(70.1)	(35.9)	(30.3)	(31.7)	(43.4)
D	PPT, 6 mm	Mean	171.4	155.7	142.9	149.0	112.8	104.4
		SD	(57.5)	(72.0)	(36.4)	(31.0)	(31.5)	(37.6)
E	PPT, 3 mm	Mean	185.9 ^a^	160.3	153.4 ^c^	164.8 ^d^	116.2	112.5 ^g^
		SD	(65.4)	(81.7)	(40.9)	(38.3)	(37.2)	(42.9)
F	Lunatec, 6 mm	Mean	168.6	152.3	142.0	149.2	111.4	105.6
		SD	(66.0)	(69.1)	(39.4)	(34.7)	(31.4)	(43.1)
G	Vepur/Poron, 7 mm	Mean	175.7 ^b^	157.0	144.4	157.7 ^e^	116.6	105.0
		SD	(58.0)	(72.7)	(36.5)	(38.2)	(38.8)	(41.4)
H	Vipod/DiaPod, 6 mm	Mean	174.8	160.5	148.1	152.6	119.0 ^f^	113.9 ^h^
		SD	(51.5)	(69.0)	(35.0)	(27.0)	(28.6)	(36.8)
	F P Effect size		6.278 <0.001 0.147	3.445 0.007 0.081	4.977 0.002 0.113	15.324 <0.001 0.282	3.426 0.024 0.081	7.865 <0.001 0.168

Note: ROI = region of interest = primary region for offloading, based on most recent plantar ulcer location or location with the highest barefoot pressure. Test: ANOVA with repeated measures with a Greenhouse–Geisser correction. Statistically significant findings in post hoc analyses with Bonferroni correction: ^a^: Significantly higher compared to B, C, D, and F. ^b^: Significantly higher compared to C. ^c^: Significantly higher compared to A, B, C, and D. ^d^: Significantly higher compared to A, B, C, D, F, and H. ^e^: Significantly higher compared to B, C, D, and F. ^f^: Significantly higher compared to C. ^g^: Significantly higher compared to C, D, and F. ^h^: Significantly higher compared to B, D, F, and G.

**Table 4:** Perceived satisfaction for shoe fit and walking comfort for the different insole top covers.

Insole	Name		Shoe Fit	Walking Comfort
A	PPT/Plastazote, 6 mm	Mean	8.1	8.0
		SD	(1.2)	(1.1)
B	PPT/P-cell, 6 mm	Mean	8.1	8.2
		SD	(1.4)	(1.3)
C	Astro form/Aero sorb, 6 mm	Mean	8.1	8.3
		SD	(1.3)	(1.4)
D	PPT, 6 mm	Mean	8.0	8.3
		SD	(1.6)	(1.2)
E	PPT, 3 mm	Mean	8.4	8.4
		SD	(1.0)	(0.8)
F	Lunatec, 6 mm	Mean	8.3	8.3
		SD	(1.3)	(1.0)
G	Vepur/Poron, 7 mm	Mean	7.8	8.0
		SD	(1.5)	(1.5)
H	Vipod/DiaPod, 6 mm	Mean	8.3	8.3
		SD	(1.4)	(1.2)

Note: Data are mean and SD scores, ranging between 1 and 10, with 10 being most satisfactory score.

**Table 5:** Follow-up measurements—difference between peak pressure at follow-up minus peak pressure at baseline—for the ROI plus the five anatomical regions that were identified as a ROI.

Insole	Name	N		ROI	Hallux	MTH1	MTH23	MTH45	Medial Midfoot
A	PPT/Plastazote, 6 mm	10	Mean	2.7	1.6	−3.8	13.2	−1.8	−0.2
			SD	12.0	19.0	15.7	25.2	12.4	8.3
B	PPT/P-cell, 6 mm	9	Mean	47.8	40.5	−2.6	28.2	14.3	3.2
			SD	68.7	69.7	38.8	50.5	44.5	30.9
C	Astro form/Aero sorb, 6 mm	9	Mean	12.7	15.3 *	−2.0	1.8	4.8	7.3
			SD	18.9	17.8	31.2	15.9	27.4	8.9
D	PPT, 6 mm	7	Mean	−2.7	−37.6 *	−4.9	7.2	0.8	−11.4 *
			SD	44.5	33.8	40.9	38.6	14.3	29.3
E	PPT, 3 mm	10	Mean	−1.9	13.8	−9.6	0.2	−7.5	−4.8
			SD	29.8	27.4	21.8	24.8	15.0	15.4
F	Lunatec, 6 mm	10	Mean	−0.7	−10.8	−5.1	9.5	4.6	−5.9
			SD	20.1	25.3	18.3	19.6	21.2	15.9
G	Vepur/Poron, 7 mm	9	Mean	20.4	19.6	−12.6	−30.0 *	−17.1	−4.6
			SD	50.4	54.3	33.2	37.4	24.3	13.5
H	Vipod/DiaPod, 6 mm	8	Mean	2.5	−11.7	−9.8	−10.6	−9.7	−4.4 *
			SD	23.1	22.8	24.7	19.0	12.8	23.1

Note: ROI = region of interest = primary region for offloading, based on most recent plantar ulcer location or location with the highest barefoot pressures. Number of cases ranges from 7–10 with some missing cases, as some top covers could not be measured at 1-month follow-up due to participant availability. Reasons were COVID-19 lockdown (*n* = 3) and technical (*n* = 1), for a total of eight top cover evaluations missing. * Significant change based on paired sample *t*-test without correction for multiple testing (*p* < 0.05).

## Data Availability

The datasets generated and analyzed during the current study are not publicly available due to current Dutch ethical legislation and the European Union GDPR Act.

## Supplementary Materials

The following supporting information can be downloaded at: https://www.mdpi.com/article/10.3390/s24175549/s1, Table S1: In-shoe peak pressures for anatomical regions not identified as ROI in the cross-sectional study; Table S2: Follow-up versus baseline in-shoe peak pressure differences for anatomical regions not identified as ROI.

## References

[1] Armstrong D.G., Tan T.W., Boulton A.J.M., Bus S.A. (2023). Diabetic foot ulcers: A review. JAMA.

[2] Bus S.A., Waaijman R., Arts M., de Haart H., Busch-Westbroek T., Van B.J., Nollet F. (2013). Effect of custom-made footwear on foot ulcer recurrence in diabetes: A multicenter randomized controlled trial. Diabetes Care.

[3] Bus S.A., Sacco I.C.N., Monteiro-Soares M., Raspovic A., Paton J., Rasmussen A., Lavery L.A., van Netten J.J. (2024). Guidelines on the prevention of foot ulcers in persons with diabetes (IWGDF 2023 update). Diabetes Metab. Res. Rev..

[4] Arts M.L., de Haart M., Waaijman R., Dahmen R., Berendsen H., Nollet F., Bus S.A. (2015). Data-driven directions for effective footwear provision for the high-risk diabetic foot. Diabet. Med..

[5] Chapman J.D., Preece S., Braunstein B., Höhne A., Nester C.J., Brueggemann P., Hutchins S. (2013). Effect of rocker shoe design features on forefoot plantar pressures in people with and without diabetes. Clin. Biomech..

[6] Guldemond N.A., Leffers P., Schaper N.C., Sanders A.P., Nieman F., Willems P., Walenkamp G.H. (2007). The effects of insole configurations on forefoot plantar pressure and walking convenience in diabetic patients with neuropathic feet. Clin. Biomech..

[7] Mueller M.J., Lott D.J., Hastings M.K., Commean P.K., Smith K.E., Pilgram T.K. (2006). Efficacy and mechanism of orthotic devices to unload metatarsal heads in people with diabetes and a history of plantar ulcers. Phys. Ther..

[8] Owings T.M., Woerner J.L., Frampton J.D., Cavanagh P.R., Botek G. (2008). Custom therapeutic insoles based on both foot shape and plantar pressure measurement provide enhanced pressure relief. Diabetes Care.

[9] van Schie C., Ulbrecht J.S., Becker M.B., Cavanagh P.R. (2000). Design criteria for rigid rocker shoes. Foot Ankle Int..

[10] Zwaferink J.B.J., Custers W., Paardekooper I., Berendsen H.A., Bus S.A. (2021). Effect of a carbon reinforcement for maximizing shoe outsole bending stiffness on plantar pressure and walking comfort in people with diabetes at high risk of foot ulceration. Gait Posture.

[11] Bus S.A., Ulbrecht J.S., Cavanagh P.R. (2004). Pressure relief and load redistribution by custom-made insoles in diabetic patients with neuropathy and foot deformity. Clin. Biomech..

[12] Bus S.A., Zwaferink J.B., Dahmen R., Busch-Westbroek T. (2020). State of the art design protocol for custom made footwear for people with diabetes and peripheral neuropathy. Diabetes Metab. Res. Rev..

[13] Collings R., Freeman J., Latour J.M., Paton J. (2021). Footwear and insole design features for offloading the diabetic at risk foot-A systematic review and meta-analyses. Endocrinol. Diabetes Metab..

[14] Brodsky J.W., Pollo F.E., Cheleuitte D., Baum B.S. (2007). Physical properties, durability, and energy-dissipation function of dual-density orthotic materials used in insoles for diabetic patients. Foot Ankle Int..

[15] Paton J., Jones R.B., Stenhouse E., Bruce G. (2007). The physical characteristics of materials used in the manufacture of orthoses for patients with diabetes. Foot Ankle Int..

[16] Nilsen F., Molund M., Lium E.A., Hvaal K.H. (2021). Material Selection for Diabetic Custom Insoles: A Systematic Review of Insole Materials and Their Properties. J. Prosthet. Orthot..

[17] Gerrard J.M., Bonanno D.R., Whittaker G.A., Landorf K.B. (2020). Effect of different orthotic materials on plantar pressures: A systematic review. J. Foot Ankle Res..

[18] Schaper N.C., van Netten J.J., Apelqvist J., Bus S.A., Fitridge R., Game F., Monteiro-Soares M., Senneville E., Board I.E. (2024). Practical guidelines on the prevention and management of diabetes-related foot disease (IWGDF 2023 update). Diabetes Metab. Res. Rev..

[19] Bus S.A., de Lange A. (2005). A comparison of the 1-step, 2-step, and 3-step protocols for obtaining barefoot plantar pressure data in the diabetic neuropathic foot. Clin. Biomech..

[20] Arts M.L., Bus S.A. (2011). Twelve steps per foot are recommended for valid and reliable in-shoe plantar pressure data in neuropathic diabetic patients wearing custom made footwear. Clin. Biomech..

[21] Waaijman R., Bus S.A. (2012). The interdependency of peak pressure and pressure-time integral in pressure studies on diabetic footwear: No need to report both parameters. Gait Posture.

[22] Nouman M., Dissaneewate T., Leelasamran W., Chatpun S. (2019). The insole materials influence the plantar pressure distributions in diabetic foot with neuropathy during different walking activities. Gait Posture.

[23] Healy A., Dunning D.N., Chockalingam N. (2012). Effect of insole material on lower limb kinematics and plantar pressures during treadmill walking. Prosthet. Orthot. Int..

[24] Rogers K., Otter S., Birch I. (2006). The effect of PORON^®^ and Plastazote^®^ insoles on forefoot plantar pressures. Br. J. Podiatry.

[25] Tong J.W., Ng E.Y. (2010). Preliminary investigation on the reduction of plantar loading pressure with different insole materials (SRP–Slow Recovery Poron^®^, P–Poron^®^, PPF–Poron^®^+Plastazote, firm and PPS–Poron^®^+Plastazote, soft). Foot.

[26] Waaijman R., Keukenkamp R., de Haart M., Polomski W.P., Nollet F., Bus S.A. (2013). Adherence to wearing prescription custom-made footwear in patients with diabetes at high risk for plantar foot ulceration. Diabetes Care.

[27] Yavuz M., Ersen A., Hartos J., Schwarz B., Garrett A.G., Lavery L.A., Wukich D.K., Adams L.S. (2017). Plantar Shear Stress in Individuals With a History of Diabetic Foot Ulcer: An Emerging Predictive Marker for Foot Ulceration. Diabetes Care.

