# Supplementary material for: Pressure-Relieving Effect of Different Insole Top Covers in People with Diabetes at High Risk of Foot Ulceration
Source: Sensors (Basel). 2024 Aug 27;24(17):5549. doi: 10.3390/s24175549 (PMC11398109; doi:10.3390/s24175549)
Supplement: Supplementary file 1 [file sensors-24-05549-s001.zip › sensors-3022106-supplementary.pdf]

Supplemental tables

Supplemental Table S1: Mean peak pressures (SD) for five anatomical regions that were not identified as ROI, for all 8 insole top covers in the cross-sectional study

Insole	Name (bottom/top)		Digiti 23	Digiti 45	Lateral midfoot	Medial heel	Lateral heel
A	PPT/Plastazote	Mean	132.5	92.0	108.3	175.8	177.1
		SD	(73.6)	(43.9)	(32.0)	(36.8)	(35.8)
B	PPT/P-cell	Mean	138.0	96.5	110.0[d]	170.5	174.3
		SD	(76.3)	(46.2)	(33.8)	(38.6)	(38.0)
C	Astro form/Aero sorb	Mean	136.4	95.8	105.7	171.9	173.4
		SD	(74.6)	(49.4)	(36.1)	(37.6)	(36.4)
D	PPT 6mm	Mean	139.3[a]	96.2	105.8	171.3	174.7
		SD	(78.3)	(46.1)	(32.7)	(36.0)	(33.4)
E	PPT 3mm	Mean	137.6	92.5	113.0[e]	184.2[g]	189.6[h]
		SD	(84.5)	(46.9)	(40.2)	(42.2)	(46.4)
F	Lunatec 6mm	Mean	138.0	95.7	106.0	175.0	175.6
		SD	(74.1)	(44.5)	(37.2)	(37.7)	(37.7)
G	Vepur/Poron	Mean	141.9	91.8	108.0	171.2	180.5
		SD	(79.1)	(45.0)	(39.6)	(36.1)	(43.4)
H	Vipod/DiaPod	Mean	145.7[b]	100.9[c]	114.1[f]	173.0	176.5
		SD	(75.5)	(47.9)	(29.5)	(37.0)	(35.1)
	F		3.022	3.518	5.865	10.577	33.0
	P		0.019	0.005	0.001	<0.001	<0.001
	Effect size		0.072	0.083	0.131	0.213	0.678

Note: ROI = region of interest = primary region for offloading, based on most recent plantar ulcer location or location with the highest barefoot pressure. Test: ANOVA with repeated measures with a Greenhouse-Geisser correction. Statistically significant findings in post-hoc analyses with Bonferroni correction: [a]: significantly higher compared to A. [b]: significantly higher compared to A and B. [c]: significantly higher compared to A and G. [d]: significantly higher compared to C, D, and F. [e]: significantly higher compared to C, D, and F. [f]: significantly higher compared to D. [g]: significantly higher compared to A, B, C, D, F, G, and H. [h]: significantly higher compared to A, B, C, D, F, and H.

Supplemental Table S2: Follow-up measurements – difference between peak pressure at follow-up minus peak pressure at baseline – for five anatomical regions that were not identified as ROI

Insole	Name	*n*		Digiti 23	Digiti 45	Lateral midfoot	Medial heel	Lateral heel
A	PPT/Plastazote	10	Mean	6.4	-4.6	-2.1	-5.7	7.8
			SD	18.6	19.2	11.5	20.1	24.8
B	PPT/P-cell	9	Mean	12.0	15.7	9.6	30.9	36.5
			SD	39.2	28.7	38.2	70.1	71.8
C	Astro form/Aero sorb	9	Mean	4.7	-6.6	15.2	-8.0*	14.4
			SD	31.0	14.6	22.7	16.7	54.9
D	PPT 6mm	7	Mean	-29.7*	-14.4*	-11.1	-7.7	-6.9
			SD	27.2	15.0	8.8	13.9	15.7
E	PPT 3mm	10	Mean	-0.9	-10.5	-2.8	-0.8	-3.0
			SD	26.0	19.3	11.6	16.3	13.3
F	Lunatec 6mm	10	Mean	-6.4	5.9	1.0	1.8	1.1
			SD	22.2	18.2	9.0	10.5	16.2
G	Vepur/Poron	9	Mean	-13.6	-7.6	-8.4	-7.7	-16.4*
			SD	42.0	16.5	16.0	17.2	21.0
H	Vipod/DiaPod	8	Mean	-8.7	-7.8	-8.5	-4.0	-1.0
			SD	26.3	15.0	9.2	16.7	14.5

Note: ROI = region of interest = primary region for offloading, based on most recent plantar ulcer location or location with the highest barefoot pressures. Number of cases ranges from 7-10 with some missing cases as some top covers could not be measured at 1-month follow-up due to participant availability. Reasons were COVID-19 lockdown (*n* = 3) and technical (*n* = 1), for a total of 8 top cover evaluations missing *significant change based on paired sample t-test without correction for multiple testing ($p < 0.05$)
